# Metabolic and Bariatric Surgery as a Strategy for Cardiovascular Risk Reduction in Obesity: A Narrative Review

**DOI:** 10.7759/cureus.112549

**Published:** 2026-07-12

**Authors:** Jan Szewczyk, Izabela Grzyb, Alicja Smolinska, Maria Boloz, Patrycja Krawczyk, Zuzanna Watek, Katarzyna Lysynkiewicz, Sylwia Haba, Ewa Dapkiewicz, Dawid Gasowski

**Affiliations:** 1 Internal Medicine, Polish Red Cross Maritime Hospital in Gdynia, Gdynia, POL; 2 Internal Medicine, Śniadeckiego Voivodeship Hospital in Bialystok, Bialystok, POL; 3 Internal Medicine, The Provincial Combined Hospital in Kielce, Kielce, POL; 4 Internal Medicine, University Clinical Hospital in Białystok, Białystok, POL; 5 Internal Medicine, Samodzielny Publiczny Zakład Opieki Zdrowotnej Ministerstwa Spraw Wewnętrznych i Administracji w Białymstoku im. Mariana Zyndrama-Kościałkowskiego, Białystok, POL; 6 Emergency Department, Samodzielny Publiczny Zakład Opieki Zdrowotnej w Łapach, Łapy, POL

**Keywords:** bariatric surgery, cardiovascular risk, metabolic surgery, obesity, weight loss

## Abstract

Obesity is a major global healthcare problem and an important contributor to cardiovascular morbidity and mortality through complex metabolic, haemodynamic, inflammatory, and endothelial mechanisms. Excess adiposity promotes the development of hypertension, dyslipidaemia, and type 2 diabetes, as well as cardiac remodelling and heart failure, significantly increasing cardiovascular risk. The aim of this review was to assess the impact of bariatric and metabolic surgery on cardiovascular risk factors and systemic changes associated with the cardiovascular system in patients with obesity. Relevant literature was identified through PubMed/MEDLINE and Google Scholar searches, focusing primarily on studies published between 2020 and 2025, including randomised controlled trials, meta-analyses, epidemiological studies, and clinical investigations. Available evidence indicates that bariatric surgery, particularly sleeve gastrectomy (SG) and Roux-en-Y gastric bypass (RYGB), induces substantial and sustained weight loss while improving key metabolic parameters. Reported benefits include significant reductions in blood pressure, improvements in glycaemic control and insulin sensitivity, favourable changes in lipid profiles, reduced systemic inflammation and oxidative stress, and improved endothelial function. Observational studies and meta-analyses further suggest that, compared with conservative treatment, bariatric surgery is associated with lower rates of major adverse cardiovascular events (MACEs), coronary artery disease (CAD), heart failure, myocardial infarction (MI), stroke, and all-cause mortality. However, bariatric procedures are associated with potential risks, such as nutritional deficiencies, postoperative hypoglycaemia, weight regain (WR), and procedure-specific complications; therefore, long-term follow-up and multidisciplinary care are required. Overall, bariatric surgery plays an important role in cardiovascular risk reduction in patients with obesity, but further prospective studies are needed to better define the durability of its benefits.

## Introduction and background

According to a systematic analysis from the Global Burden of Disease Study 2023, obesity, in particular, has become a major driver of poor health outcomes worldwide, and its prevalence varies across countries in nearly all regions [1]. The worsening obesity pandemic remains a major contributor to the development of cardiovascular diseases (CVDs). CVDs remain a leading cause of morbidity and mortality in developed countries [2]. It has been shown that severe obesity adversely affects cardiovascular function through a variety of mechanisms, including endothelial dysfunction and remodelling of small blood vessels, which increase the risk of arrhythmias, cardiomyopathy, and congestive heart failure [3]. Obesity-related comorbidities, including type 2 diabetes mellitus, CVD, and non-alcoholic liver disease, represent a major health and economic burden, with steadily increasing numbers worldwide [4].

The treatment of obesity and its associated comorbidities is challenging and is based on lifestyle changes, including diet and physical activity [5]. Pharmacological treatment can be helpful; however, despite recent progress with incretin-based therapies, limited accessibility, adverse effects, long-term adherence, and weight regain (WR) after discontinuation remain important limitations [6]. Bariatric surgery is an accepted treatment for patients with severe obesity. According to the current American Society for Metabolic and Bariatric Surgery (ASMBS) and International Federation for the Surgery of Obesity and Metabolic Disorders (IFSO) guidelines, metabolic and bariatric surgery is recommended for individuals with a BMI ≥35 kg/m², regardless of the presence or absence of obesity-associated comorbidities, and should also be considered in patients with a BMI of 30-34.9 kg/m² and metabolic disease [7]. Several bariatric procedures are currently performed, with sleeve gastrectomy (SG) and Roux-en-Y gastric bypass (RYGB) being the most common. SG is the most frequently performed procedure, accounting for approximately 65-80% of cases [8]. Advances in surgical techniques, particularly laparoscopic approaches, have resulted in a low incidence of serious complications and a 30-day mortality rate of <0.5%. However, other adverse effects of bariatric surgery remain evident, such as dumping syndrome [5]. Following surgical intervention, the clinical profile of patients with CVD undergoes significant changes; whilst some of these changes have beneficial effects, others require additional care.

The aim of this review was to assess the impact of metabolic surgery on systemic changes associated with the cardiovascular system.

## Review

Review methodology

A narrative review of the available literature was conducted. References for the review were identified through searches of PubMed/MEDLINE and Google Scholar for articles published between 2020 and 2025, although older references were used when appropriate. The online searches were conducted independently by four investigators. We used the search terms “bariatric surgery” and “metabolic surgery” in combination with the terms “obesity”, “cardiovascular risk”, “hypertension”, “insulin resistance”, and “dyslipidemia”. Publications, including randomized controlled trials, meta-analyses, epidemiological studies, and experimental studies, were analysed. The initial search identified 289 articles across all databases. After the removal of duplicates, 255 articles were reviewed.

Obesity and cardiovascular risk

Obesity has been recognised as one of the major factors contributing to increased cardiovascular risk [3]. It promotes the development of CVD through both direct and indirect mechanisms; the pathophysiological mechanisms involved in obesity-related dysmetabolic conditions are complex and multifactorial. The general conclusion is that both indirect obesity-induced metabolic comorbidities, such as diabetes, hypertension (HTN), and dyslipidemia, and direct vascular dysfunction and injury caused by excess adiposity significantly contribute to coronary atherosclerosis and ischaemic heart disease [3].

Structural Remodelling, Inflammation, and Adiposopathy

In individuals with obesity, total blood volume and cardiac output are increased due to the Frank-Starling mechanism, resulting in cardiovascular overload and, consequently, structural and functional changes such as left ventricular hypertrophy (LVH). LVH is commonly associated with elevated left ventricular filling pressure, left ventricular diastolic dysfunction, and a predisposition to HF [9]. Moreover, severe obesity may contribute to obesity-related cardiomyopathy, sometimes referred to as adipositas cordis, through haemodynamic overload, myocardial lipid accumulation, fatty infiltration of the myocardium, and inflammation [10].

Another factor directly linking obesity and CVD is adipose tissue dysfunction. Under various physiological and pathological conditions, adipocytes secrete hormones and peptides known as adipokines; these play an important role in the local and systemic regulation of energy homeostasis and inflammation [11]. In individuals with obesity, a condition called adiposopathy occurs; it involves functional changes in adipose tissue, such as adipocyte hypertrophy, impaired adipogenesis, increased free fatty acid release, endocrinopathies including hypoadiponectinemia and hyperleptinemia, and proinflammatory cytokine secretion. These changes are ultimately associated with dysfunctional fat accumulation and increased cardiovascular risk [12]. Epicardial adipose tissue-derived adipocytokines, inflammation, and systemic mediators such as reactive oxygen species may favour the development of a local proatherogenic environment, thereby promoting the pathogenesis of coronary artery disease (CAD) [13]. In addition to affecting larger arteries, excess adiposity is also linked to endothelial dysfunction and pathological remodelling of the coronary microvasculature [14].

Hypertension, Dyslipidemia, and Diabetes

Studies have shown a nearly linear correlation between BMI and systolic blood pressure, and it has been suggested that approximately 78% of primary HTN in men and 65% in women may be attributable to excess weight gain [15]. Several mechanisms contribute to the development of HTN in individuals with obesity, including: (1) physical compression of the kidneys by visceral fat, leading to increased intrarenal pressure and impaired natriuresis; (2) activation of the renin-angiotensin-aldosterone system via mineralocorticoid receptors; and (3) activation of the sympathetic nervous system [9]. Patients with obesity and HTN have been reported to have increased left ventricular mass (LVM) and higher stroke volume (SV) and cardiac output (CO) than individuals with obesity whose blood pressure is within the normal range [16].

Dyslipidemia is a well-known risk factor for and contributor to CVD. In individuals with obesity, elevated levels of total cholesterol (TC) and triglycerides (TG), as well as low levels of high-density lipoprotein cholesterol (HDL-C), may be observed. Interestingly, low-density lipoprotein cholesterol (LDL-C) is frequently found to be within the normal range in the majority of patients with obesity [17]. It has been reported that treating these lipid abnormalities may reduce the risk of CVD by 30% over a five-year period [16]. A direct causal role of dyslipidemia in the pathogenesis of atherosclerotic cardiovascular disease (ASCVD) is well established. An excessive number of plasma LDL particles promotes foam-cell formation, which contributes to the development of atherosclerotic plaques. An occlusive vascular event may occur when a plaque ruptures, leading to myocardial infarction (MI), stroke, or peripheral limb ischaemia [18]. Epidemiological studies have also shown low HDL-C levels and elevated TG levels to be independent risk factors for ASCVD [19]. Moreover, elevated TG levels may indirectly promote proatherogenic mechanisms such as inflammation, thrombogenesis, and abnormal endothelial function [20].

Obesity is an important driving factor in the development of type 2 diabetes. Ectopic and visceral adiposity have been linked to insulin resistance, a potential mediating factor linking obesity, type 2 diabetes, and cardiovascular risk [13]. The metabolic syndrome, a cluster of conditions including abdominal obesity, HTN, glucose metabolism disorders, dyslipidemia, and insulin resistance, has been recognised as a risk factor for cardiovascular morbidity and mortality [13]. Moreover, the presence of the metabolic syndrome has been shown to increase the risk of developing cardiac dysfunction and HF [21]. In the ongoing Uppsala Longitudinal Study of Adult Men, the presence of the metabolic syndrome was associated with a more than threefold increase in the risk of developing HF during a 20-year follow-up period [22].

Bariatric surgery procedures

The high success rate of surgical treatment for obesity has led to the development and recent revision of guidelines aimed at optimising the outcomes of bariatric and metabolic surgery [7]. According to the 2022 ASMBS and IFSO guidelines, bariatric surgery is recommended for patients with a BMI ≥35 kg/m², irrespective of the presence of comorbidities such as diabetes or HTN. Surgery should also be considered in patients with a BMI of 30-34.9 kg/m² and coexisting metabolic disease, particularly type 2 diabetes or other obesity-related complications. Bariatric surgery may also be performed in children and adolescents, provided that established eligibility criteria are met. In Asian populations, BMI thresholds should be adjusted downwards because of the increased metabolic risk; a BMI ≥25 kg/m² suggests clinical obesity, and individuals with a BMI ≥27.5 kg/m² should be offered metabolic and bariatric surgery when appropriate [7]. The revision of these guidelines has also prompted updates to national recommendations. The Polish Expert Consensus on Metabolic and Bariatric Surgery: 2025 Update allows patient eligibility to be determined based on the highest recorded BMI [23].

Sleeve Gastrectomy

SG accounts for approximately 65-80% of bariatric procedures, making it one of the most commonly performed techniques worldwide. The procedure involves the resection of approximately 75-80% of the stomach along the greater curvature, resulting in a tubular gastric remnant with a capacity of 60-100 mL. This induces a range of metabolic changes that contribute to weight loss. The observed weight reduction is driven by both mechanical restriction due to decreased gastric capacity and hormonal alterations. SG is associated with reduced ghrelin secretion, accelerated gastric emptying, and altered incretin hormone release [24].

Roux-en-Y Gastric Bypass

In RYGB, a small gastric pouch of 15-30 mL is created from the proximal stomach and anastomosed to the jejunum via a gastrojejunostomy. This procedure bypasses a substantial portion of the stomach and proximal small intestine, thereby restricting food intake and reducing nutrient absorption [24].

Metabolic Mechanisms and Effects of Bariatric Surgery

The metabolic effects of bariatric surgery extend beyond simple caloric restriction and involve complex hormonal and neuroendocrine mechanisms. The most straightforward explanation for weight loss is mechanical restriction, as the reduced gastric volume limits food intake, leading to weight reduction and improved metabolic parameters. However, numerous studies indicate that weight loss is largely driven by physiological adaptations that occur in response to surgery.

Bariatric surgery significantly alters the secretion of gut hormones, thereby influencing appetite and glucose metabolism. In particular, levels of glucagon-like peptide-1 (GLP-1) and peptide YY (PYY) increase markedly after surgery, which is associated with enhanced satiety and improved insulin secretion. A reduction in ghrelin levels is also observed. These hormonal mechanisms are considered key contributors to the remission of type 2 diabetes, among other factors [25]. Bariatric surgery also influences gut microbiota composition and bile acid metabolism, further supporting metabolic improvement. Rapid weight loss following bariatric surgery may also be associated with an increased risk of gallstone formation. Collectively, these mechanisms lead to reductions in blood glucose and TG levels and improvements in insulin sensitivity. Consequently, bariatric surgery significantly reduces cardiovascular risk and may enable the reduction or discontinuation of pharmacological treatment, including antihypertensive therapy [26].

The impact of bariatric surgery on cardiovascular risk factors

Mentias A et al. conducted a study with a median follow-up of four years evaluating the long-term outcomes of bariatric surgery in a Medicare population. The study cohort included 189,770 patients matched for age, sex, and degree of obesity. Among the 94,885 patients who underwent bariatric surgery, 62,101 (65.5%) underwent SG, 31,546 (33.3%) underwent gastric bypass, and 1,238 (1.3%) underwent gastric banding. Bariatric surgery was associated with a 37% reduction in all-cause mortality, with mortality rates of 9.2 versus 14.7 per 1,000 person-years. Additionally, significant reductions in the risks of cardiovascular outcomes were observed, including a 54% lower risk of new-onset HF, a 37% lower risk of MI, and a 29% lower risk of ischaemic stroke [27]. After applying instrumental variable analysis (IVA), aimed at minimising the effects of confounding factors, the authors reported an even greater benefit, with a 60% reduction in all-cause mortality, a 79% reduction in new-onset HF, and an 80% reduction in MI compared with the control group. No significant difference in stroke risk was observed [27].

Chandrakumar H et al. conducted a meta-analysis including 49 studies evaluating the effects of bariatric surgery on cardiovascular outcomes and mortality. The analysis demonstrated a reduced risk of CAD (hazard ratio: 0.68), as well as reduced risks of MI, HF, cerebrovascular accident (CVA), and cardiovascular mortality. These benefits were observed across both younger and older populations, suggesting broad clinical applicability [28]. HTN remission has been reported in up to 68.1% of patients within the first year following surgery. However, insufficient weight loss during the first postoperative year may increase the risk of recurrence over a three-year follow-up period, with approximately 21.9% of patients experiencing relapse [29].

Compared with pharmacological treatment alone, patients undergoing bariatric surgery achieve superior outcomes, including reductions in blood pressure and body weight and improvements in glycaemic control. Both systolic and diastolic blood pressure are significantly reduced [30]. Among surgical techniques, gastric bypass generally demonstrates superior long-term efficacy compared with SG, particularly in achieving sustained blood pressure control. Bariatric surgery also leads to significant improvements in lipid profiles. Between one month and four years following RYGB, substantial reductions in LDL-C and TG levels, along with increases in HDL-C levels, have been observed [31].

In a cohort study [32], levels of IL-6, leptin, oxidised LDL (oxLDL), and adiponectin were assessed. An analysis of 52 patients enrolled in two clinical trials demonstrated that RYGB significantly reduced IL-6, leptin, and oxLDL levels while increasing adiponectin concentrations. These findings suggest that bariatric surgery exerts beneficial effects through the modulation of inflammation and oxidative stress, which may also contribute to reductions in the risks of type 2 diabetes and CAD.

Jamialahmadi T et al. conducted a meta-analysis evaluating the impact of bariatric surgery on endothelial function. Based on 23 clinical trials involving 891 patients, a significant improvement in flow-mediated dilation (FMD) was observed, with progressive improvement over time. In addition to improved endothelial function, significant reductions in blood pressure and body weight were also reported [33].

Verrastro O et al. conducted the randomised BRAVES trial (Bariatric-Metabolic Surgery vs. Lifestyle Intervention Plus Best Medical Care) at three hospitals in Rome. The primary endpoint, defined as the histological resolution of NASH without worsening of fibrosis, was achieved by 54 of 96 patients (54%) in the RYGB group and 55 of 96 patients (55%) in the SG group, compared with 15 of 96 patients (15%) in the lifestyle intervention group. The calculated probability of NASH resolution was 3.60 times greater (95% CI: 2.19-5.92; p < 0.0001) in the RYGB group and 3.67 times greater (95% CI: 2.23-6.02; p < 0.0001) in the RYGB group compared with the lifestyle intervention group. As NASH is associated with an increased risk of CVD and death, these findings may have implications for cardiovascular risk; however, the study did not directly assess cardiovascular outcomes [34].

Bariatric surgery has also been shown to significantly reduce the risk of major adverse cardiovascular events (MACE) compared with conservative management, including dietary and pharmacological interventions [35]. Bariatric surgery has a significant and multifactorial impact on cardiovascular risk reduction. Available observational studies and meta-analyses indicate decreased risks of all-cause mortality, coronary events, HF, and stroke compared with conservative treatment. These benefits are primarily driven by improvements in key cardiovascular risk factors, including sustained weight loss, better glycaemic control, reduced blood pressure, and favourable changes in lipid profiles. In addition, reductions in systemic inflammation and oxidative stress and improvements in endothelial function have been observed. Overall, the evidence suggests that bariatric surgery not only provides durable weight reduction but also plays an important role in CVD prevention and in reducing mortality among patients with obesity.

Limitations and potential risks

Despite the effectiveness of bariatric surgery, it has inherent limitations and potential risks that must be carefully considered. Each bariatric procedure carries specific metabolic complications that may result in nutritional deficiencies and other complications. SG is associated with complications such as staple-line leak, bleeding, gastro-oesophageal reflux, and nutritional deficiencies, whereas RYGB is associated with anastomotic complications, marginal ulceration, internal hernia, small bowel obstruction, dumping syndrome, and micronutrient deficiencies. Data from the US National Patient-Centered Clinical Research Network (33,560 adults) show that interventions, reoperations, and hospitalizations are relatively common after bariatric surgery, with higher rates observed after RYGB compared to SG [36].

Bariatric surgery alters gastrointestinal anatomy, affecting digestion and nutrient intake. Patients typically eat smaller portions and may experience changes in taste, while symptoms such as vomiting and diarrhea can further reduce intake. Together, these changes increase the risk of nutrient deficiencies [37]. Reduced gastric acid after surgery further limits nutrient absorption. Although the extent varies by procedure, bariatric surgery is associated with lower levels of iron, vitamin B1, folate, vitamin B12, vitamin D, and calcium; more malabsorptive procedures may also increase the risk of fat-soluble vitamin and protein deficiencies. Adherence to supplementation and regular follow-up are therefore essential for maintaining nutritional status [38].

It is now well established that a large proportion of patients experience significant weight regain (WR) due to not following strict diet advice, although its prevalence varies widely depending on the definition and time since surgery [39]. Anatomical and surgical factors account for only a small proportion of cases; most WR appears to result from increased post-operative caloric intake driven by heightened appetite and maladaptive or dysregulated eating behaviours, reduced physical activity, and psychosocial stressors. However, lifestyle interventions have shown limited efficacy. Revisional surgery may be effective in selected cases but carries higher risk. Similarly, dietary, behavioural, and exercise-based strategies have shown little to no meaningful effect in reversing WR. Evidence on pharmacotherapy is limited, largely consisting of retrospective studies and small open-label trials; therefore, the effectiveness of anti-obesity medications in post-bariatric patients requires further investigation [40].

One common complication of bariatric surgery is hypoglycemia, which may occur months to years postoperatively and can significantly affect quality of life. There are no medications currently approved specifically for this condition. Reported prevalence varies, with symptoms observed in up to one-third of patients during mixed-meal testing and incidence ranging from 9.1% to 32.8% on oral glucose tolerance testing [41]. Recurrent hypoglycaemia may contribute to reduced quality of life, increased risk of accidents and cardiovascular events, and potential WR due to compensatory overeating. Therefore, management includes dietary modification and available pharmacological options, with consideration of continuous glucose monitoring (CGM) [42].

As this is a review, the conclusions are limited by the quality of the existing literature. The available evidence is highly heterogeneous, particularly with regard to the specific surgical procedure used, baseline patient characteristics (such as degree of obesity and metabolic comorbidities), length of follow-up, and the definitions used for outcomes including weight loss, WR, and metabolic improvement. In addition, many studies rely on indirect or non-standardised outcome measures, which reduce comparability and may affect the veracity of interpretations. Important influencing factors, such as dietary adherence, physical activity, psychological status, and long-term compliance with supplementation and clinical follow-up, are frequently underreported or inconsistently accounted for in the literature.

Differences in the anatomical and physiological effects of various bariatric procedures further complicate interpretation, as they can differentially influence nutrient absorption, hormonal regulation, and biochemical parameters. Taken together, these issues highlight the need for well-designed, prospective, and standardised studies to more accurately characterise long-term outcomes after bariatric surgery.

## Conclusions

Obesity significantly increases cardiovascular risk through metabolic, haemodynamic, inflammatory, and endothelial mechanisms. It contributes to HTN, dyslipidaemia, insulin resistance, type 2 diabetes, endothelial dysfunction, cardiac remodelling, and heart failure. Bariatric surgery is an effective treatment for patients with severe obesity, and its benefits extend beyond weight reduction. Available evidence indicates that it improves body weight, blood pressure, glycaemic control, and lipid profiles while also reducing inflammation, oxidative stress, and endothelial dysfunction. Observational studies and meta-analyses suggest that bariatric surgery is associated with lower risks of all-cause mortality, coronary events, heart failure, and major adverse cardiovascular events compared with conservative treatment. However, bariatric surgery requires long-term follow-up because of possible complications, nutritional deficiencies, post-bariatric hypoglycaemia, and WR. Further prospective studies are needed to better define the durability of its benefits.
